# Report of the Pennsylvania Hospital for the Insane, for the Year 1842

**Published:** 1843-03-04

**Authors:** 


### BIBLIOGRAPHICAL NOTICES.

*Report of the Pennsylvania Hospital for the Insane, for the year* 1842. By Thomas S. Kirkbride, M. D., Physician to the Institution. Philadelphia: 1843. 8vo. pp. 50.

This is a most interesting and instructive Report, and highly creditable to the source from whence it emanates. The Pennsylvania Hospital for the Insane is now completed, and fully organized.

"It is situated about two miles west of Philadelphia, on a fine farm of 111 acres of fertile and undulating land, upon which are several groves of fine forest trees, and streams of running water; its buildings, (with the pleasure ground, garden, and deer-park, comprising 41 acres,) are surrounded by a substantial stone wall, more than a mile long, and ten and a half feet high,—so admirably arranged, that while it is scarcely seen from the hospital—it affords that perfect quiet and privacy which could not otherwise be obtained, and without interfering with the views of the fine scenery and stirring objects beyond it. The hospital—imposing in appearance—fire-proof—and constructed in the most durable manner, contains 204 chambers for patients and their attendants,—numerous handsome and well furnished parlours,—spacious and extensive corridors,—abundant means for ventilation and warming the whole with heated air—a bountiful supply of water in every part, and numerous fixtures and arrangements, specially intended for the comfort and treatment of its inmates. To these may be added, the buildings necessary for conducting the farm—for the residence of several engaged on the premises, and a fine workshop for the employment of the patients."

All this, too, has been done from a fund especially created for the purpose.

Passing over many interesting details, for which we refer our readers to the Report itself, which show an increase in the number of admissions, and a decided gain in the number of cures and discharges, we shall pass to the subject of treatment. The importance of early treatment is strongly and properly urged. In addition to its propriety on the score of humanity, much is gained in economy, and individuals and communities are both largely benefitted. This statement is confirmed by reference to the register of the institution.

The avoidance of deception in the treatment of the insane is insisted on, and the inconveniences and impropriety of a contrary course fairly stated.

An outline of the treatment pursued in the Hospital is given, and is in strict accordance with that most generally adopted in the best institutions abroad and in our own country, and one calculated to inspire general confidence, and dissipate any prejudices which may exist on the part of the relatives of those who are thus afflicted.

The *moral* treatment, all important in cases of insanity, receives every attention in all its varieties and details. Free and systematic exercise in the open air, working at some trade, regular labour on the farm, &c.—all serve to calm nervous excitability, and break up a vicious train of thought.

"Next in importance to these means, and available for a much larger number of those who enter this institution, is regular daily exercise, by long walks in the country; always, if possible, for some definite object, as a view of fine scenery—the examination of some public work, or the inspection of a manufactory. Our proximity to the city of Philadelphia has afforded us an almost exhaustless variety in these objects to which those who were suitable, have resorted during the entire year. Almost every point of interest within four or five miles has been frequently visited,—not only preventing the monotony of the parlors and halls, but offering to our patients interesting objects and subjects for pleasant reflection afterwards. A great variety of public works, finished and in progress—numerous manufacturing establishments—the Mint—Girard College—the Water and Gas Works—the Institutions for the Blind, and for the Deaf and Dumb—the Navy Yard, and the Hospital in the city, are only a few among the points that have been reached by our walking parties. Many of the exhibitions in the city—some lectures and concerts, and, occasionally, a public meeting, have also been attended.

"Many of the patients have full liberty to walk wherever their pleasure leads them, but an attendant generally accompanies a walking party. I do not recollect, except, perhaps, in one or two instances, the slightest violation of the confidence implied by these indulgences. There are some patients whose promises are as good as any guard that can be placed about them, and the violation of pledges among the insane, is much more rare than might be expected."

When the mental and physical condition of the patient forbids the excursions, exercise is taken within the inclosure, in a carriage, or on the pleasure railroad. The library is in constant demand, especially the periodical department.

"Writing—drawing—painting—the study of the mathematics, and other branches of learning, have tended to beguile many tedious hours. Several gentlemen have been usefully engaged in imparting instruction to others in the same ward, and two have been improved by giving regular lessons, for a short time, in one of the modern languages."

Several patients have been benefitted by associating with and instructing others. Games and music fill up many vacant hours, and are much enjoyed.

During the past summer a concert was given in the large hall of the centre building, and was attended by seventy patients, who were all delighted. In the last volume of this journal we gave an account of an interesting exhibition at Bicêtre, the insane hospital near Paris, whioh many of our readers will doubtless recollect. One of the leading pupils of the Conservatoire is a resident within its walls, and already much progress has been made in music.

On Christmas and New Year's eve parties were given, which passed off pleasantly and merrily. By the last report of the Hanwell Asylum, near London, the largest insane asylum in Great Britain, containing about a thousand patients, under the care of Dr. Conolly, we find that on New Year's eve nearly 300 female patients participated in an entertainment, and that among these were nineteen patients, who, under the old system, were always in restraint. The matron's birth-day was celebrated, too, by a general fete.

Restraint is nearly banished from the code of the institution, and during the last year no restraining apparatus has been used, except in two cases. The only indication recognized for its use is the benefit or safety of the patient. In all, or nearly all, the insane asylums of Great Britain it has been abandoned. Dr. Conolly, in the Report just alluded to, thus records his deliberate opinion, "at first encouraged, with much diffidence," that "the management of a large asylum is not only practicable without the application of bodily coercion to the patients, but that, after the total disuse of such method of control, the whole character of an asylum undergoes a gradual and beneficial change."

We regret that our limits do not enable us to do more justice to the admirable system pursued in this well-conducted institution. The perusal of the report will repay all who delight in the holy triumph of genuine philanthrophy, and all will be ready to exclaim in the language of Dr. Conolly, who has done, himself, so much towards the establishment of the system of non-restraint, that

"Insanity, thus treated, undergoes great if not unexpected modifications, and the wards of lunatic asylums no longer illustrate the harrowing description of their former state. Mania, not exasperated by severity, and melancholia, not deepened by the want of all ordinary consolations, lose the exaggerated character in which they were formerly beheld. Hope takes the place of fear; serenity is substituted for discontent, and the mind is left in a condition favorable to every impression likely to call forth salutary efforts. A chance is thus afforded, to every impaired mind, of recovery to an extent only limited by causes which no human art can remove."

